# Circulating exosomal microRNAs as potential biomarkers of hepatic injury and inflammation in a murine model of glycogen storage disease type 1a

**DOI:** 10.1242/dmm.043364

**Published:** 2020-09-18

**Authors:** Roberta Resaz, Davide Cangelosi, Martina Morini, Daniela Segalerba, Luca Mastracci, Federica Grillo, Maria Carla Bosco, Cristina Bottino, Irma Colombo, Alessandra Eva

**Affiliations:** 1 Laboratory of Molecular Biology, IRCCS Istituto Giannina Gaslini, Via G. Gaslini 5, 16147 Genova, Italy; 2 Department of Surgical and Diagnostic Sciences (DISC), Anatomic Pathology Unit, Università degli Studi di Genova, Viale Benedetto XV 6, 16132 Genova, Italy; 3 National Cancer Research Institute, IRCCS Ospedale Policlinico San Martino, Largo Rosanna Benzi 10, 16132 Genova, Italy; 4 Department of Experimental Medicine, School of Medicine, Università degli Studi di Genova, Via L. B. Alberti 2, 16132 Genova, Italy; 5 Laboratory of Clinical and Experimental Immunology, IRCCS Istituto Giannina Gaslini, Via G. Gaslini 5, 16147 Genova, Italy; 6 Department of Pharmacological and Biomolecular Sciences, Università degli Studi di Milano, via D. Trentacoste 2, 20134 Milano, Italy

**Keywords:** Glycogen storage disease type 1a, Hepatocellular adenoma, Biomarkers, Exosomes, Liver, MicroRNA

## Abstract

Most patients affected by glycogen storage disease type 1a (GSD1a), an inherited metabolic disorder caused by mutations in the enzyme glucose-6-phosphatase-α (G6Pase-α), develop renal and liver complications, including the development of hepatocellular adenoma/carcinoma. The purpose of this study was to identify potential biomarkers of the pathophysiology of the GSD1a-affected liver. To this end, we used the plasma exosomes of a murine model of GSD1a, the LS-*G6pc*^−/^^−^ mouse, to uncover the modulation in microRNA expression associated with the disease. The microRNAs differentially expressed between LS-*G6pc*^−/−^ and wild-type mice, LS-*G6pc*^−/−^ mice with hepatocellular adenoma and LS-*G6pc*^−/−^ mice without adenoma, and LS-*G6pc*^−/−^ mice with amyloidosis and LS-*G6pc*^−/−^ mice without amyloidosis were identified. Pathway analysis demonstrated that the target genes of the differentially expressed microRNA were significantly enriched for the insulin signaling pathway, glucose and lipid metabolism, Wnt/β-catenin, telomere maintenance and hepatocellular carcinoma, and chemokine and immune regulation signaling pathways. Although some microRNAs were common to the different pathologic conditions, others were unique to the cancerous or inflammatory status of the animals. Therefore, the altered expression of several microRNAs is correlated with various pathologic liver states and might help to distinguish them during the progression of the disease and the development of late GSD1a-associated complications.

## Abstract (precis)

**Summary:** Mice with glycogen storage disease type 1a (GSD1a) show altered expression of several microRNAs, correlated with various pathologic liver states. This might help us to understand the progression of the disease and the development of late GSD1a-associated complications.

## INTRODUCTION

Glycogen storage disease type 1a (GSD1a) is an autosomal rare metabolic disorder caused by a mutation in the catalytic subunit of glucose-6-phosphatase-α (G6Pase-α), a key enzyme in glucose homeostasis. G6Pase-α is expressed in the liver, kidney and intestine, and catalyzes the hydrolysis of glucose-6-phosphate (G6P) to glucose and phosphate, the terminal steps in gluconeogenesis and glycogenolysis (Chou et al., 2002).

Patients with GSD1a are unable to maintain glucose homeostasis and show growth retardation, hypoglycemia, hepatomegaly, kidney enlargement, hyperlipidemia, hyperuricemia and lactic acidemia. Long-term symptoms include gout, osteoporosis, renal failure and hepatic adenomas, with risk for malignancy. The disease is controlled by dietary therapies, which consist of continuous nasogastric infusion of glucose or frequent oral administration of uncooked cornstarch (Chen et al., 1984), to prevent hypoglycemia.

Unfortunately, the control of hypoglycemia cannot prevent the progressive deterioration of the liver and kidney. Liver dysmetabolism is severe, and blood chemistry parameters are altered, with high triglyceride and cholesterol concentrations. Persistent liver dysmetabolism results in a progressive worsening of the clinical parameters and the formation of hepatocellular adenomas (HCAs), which progress to hepatocellular carcinomas (HCCs) in 10% of cases (Labrune et al., 1997). Fifty-two percent of GSD1a adenomas can be classified as inflammatory type (IHCA) and 28% as β-catenin-mutated type with upregulation of glutamine synthetase (bHCA), and the remaining 20% are unclassified (UHCA) (Calderaro et al., 2013). The elevated percentage of adenomas with β-catenin-activating mutations might explain the risk of malignant transformation of HCA to HCC in GSD1a patients. Liver transplant is the only option in the most severe cases.

GSD1a patients exhibit marked variability in the severity of symptoms and complications, and the underlying pathologic pathways that develop with the progress of the disease are poorly understood. MicroRNAs are small, noncoding RNAs that regulate gene expression by targeting messenger RNA and are actively studied as biomarkers for their stability, as diagnostic/prognostic indicators for their mirroring cellular components and as a source of indication of therapeutic targets for their biological activities. Aberrant microRNA expression profiles have been reported in cancer, rare diseases and tissue degeneration (Hu et al., 2012).

The use of genetically engineered mouse models can be an efficient way of discovering prognostic markers and can minimize the problems associated with the use of human subjects, such as the variability in the histopathologic subtypes of the subjects enrolled in the study or the lack of homogeneous methods for sample collection and storage, because we can control the age of the mice, the environmental factors and the sampling protocol.

In this study, we explored the possibility of using exosomal microRNAs (Exo-miRs) as disease markers in a liver-specific murine model of GSD1a, LS-*G6pc*^−/−^, that we have generated (Resaz et al., 2014). Our animal model, in which only the liver is affected, is ideal to identify the contribution of Exo-miRs to the specific hepatic pathologic manifestations of GSD1a. We analyzed the expression of Exo-miRs in the plasma of LS-*G6pc*^−/−^ mice to derive specific biomarkers and prognostic indicators of liver degeneration, onset of HCA and its progression to HCC. We identified potential biomarkers of the pathophysiology of the diseased liver, including Exo-miRs discriminating LS-*G6pc*^−/−^ mice with adenomas from LS-*G6pc*^−/−^ mice without adenomas. Our results indicate the potential of blood as a surrogate tissue to study the development of HCA and malignant transformation to HCC in GSD1a patients.

## RESULTS

### Analysis of Exo-miR and data normalization

We searched for Exo-miR biomarkers for pathologic manifestations of GSD1a in plasma exosomes of LS-*G6pc*^−/−^ mice of different ages by comparing the Exo-miR expression profiles of diseased versus control wild-type (WT) mice. A flowchart summarizing the main steps of these analyses is shown in Fig. S1. Mice were divided into six groups according to their age. A total of 45 LS-*G6pc*^−/−^ and 18 WT mice were analyzed. All LS-*G6pc*^−/−^ mice used in the study displayed the features typical of this mouse model of GSD1a (Resaz et al., 2014). Sixteen LS-*G6pc*^−/−^ mice developed HCA. None of the WT mice showed liver abnormalities, as expected. Exosomes from 100 µl of plasma of LS-*G6pc*^−/−^ mice and age-matched controls were isolated. Total RNA was extracted, and the presence of Exo-miRs was evaluated by capillary electrophoresis to ensure the retrieval of sufficient material for the subsequent analyses. RNA samples were reverse transcribed, pre-amplified and used to set up a rodent microRNA array card that allowed measurement of the expression of 381 targets for each sample by qRT-PCR.

Raw qRT-PCR expression data were analyzed with the PIPE-T tool to remove any unwanted technical variability, filtering out Exo-miRs for which expression was not sufficiently reliable and handling the missing values that occurred in the raw data profiles (see Materials and Methods). The raw data were normalized using the global mean method (Mestdagh et al., 2009), which was effective in reducing unwanted technical variability (Fig. S2A,B). Noise reduction was significant (Kolmogorov–Smirnov *P*-value <0.05), as shown in Fig. S2C. Given that missing values are not uncommon when microRNA expression values are analyzed, we measured their proportion in LS-*G6pc*^−/−^ and WT profiles and found that 11,769 of 24,192 *Ct* values (48.6%) were missing. Given that missing values are difficult to handle using the standard statistical analysis, it was necessary to filter out those Exo-miRs for which the number of missing values exceeded 5% of the number of samples and to impute the remaining missing values. We used the Mestdagh method for imputing missing values. After filtering and imputation, we obtained a total number of 61 Exo-miRs to be considered for further analysis (data not shown).

### LS-*G6pc*^−/−^ mice express deregulated Exo-miR

We compared Exo-miR expression levels in groups of mice characterized by various pathologic conditions to identify deregulated Exo-miRs or an Exo-miR signature specific for the evolution of the disease. Initially, we compared LS-*G6pc*^−/−^ mice with WT mice. Our analysis identified 11 downregulated and one upregulated Exo-miR in LS-*G6pc*^−/−^ mice in comparison to WT mice (Table 1). Of these, several, including miR-29amiR-145a-5p, miR-342-3p, miR-744-5pmiR-15b-5p and miR-142-3p, are considered biomarkers of HCC and are involved in HCC growth, metastasis or resistance to chemotherapy (Chen et al., 2015; Fu et al., 2018; Gao et al., 2017; Li et al., 2017; Liu et al., 2018; Mahati et al., 2017; Qiu et al., 2019; Shi et al., 2015; Tan et al., 2015; Tsang et al., 2015; Wang et al., 2017). Moreover, the downregulated miRs let-7i-5p, miR-29-3p, miR-342-3p and miR-744-5p have been associated with signaling pathways relevant in glucose and lipid metabolism (Cheng et al., 2018; Li et al., 2013; Liang et al., 2013; Zhang et al., 2019b; Zhu et al., 2011).

**Table 1.**
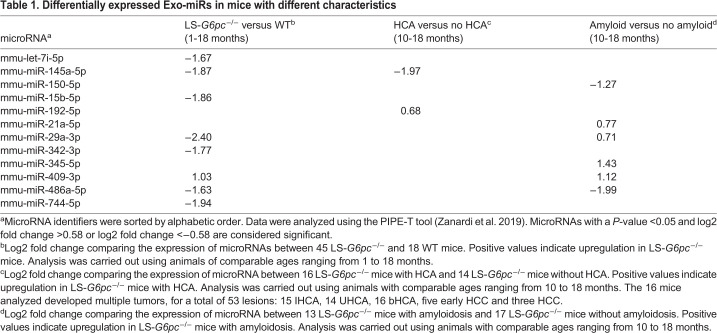
**Differentially expressed Exo-miRs in mice with different characteristics**

We then compared Exo-miR expression levels in LS-*G6pc*^−/−^ mice with HCA versus LS-*G6pc*^−/−^ mice without HCA. Differential expression analysis by the rank product method identified one downregulated and one upregulated Exo-miR in LS-*G6pc*^−/−^ mice with HCA versus LS-*G6pc*^−/−^ mice without HCA (Table 1). Of note, miR-145a-5p was also found to be downregulated in LS-*G6pc*^−/−^ mice compared with WT (Table 1).

We previously reported that 90% of LS-*G6pc*^−/−^ mice show marked amyloid deposition in the liver and kidney by 12 months of age (Resaz et al., 2014). Amyloidosis is a complication of GSD, especially type 1b, usually with a renal localization (Dick et al., 2012). The reason for this is not clear, but it is reasonable to speculate that amyloidosis in LS-*G6pc*^−/−^ mice might be associated with liver inflammation. Thus, we compared Exo-miR expression levels in LS-*G6pc*^−/−^ mice with and without amyloidosis. Differential expression analysis identified two downregulated Exo-miRs and five upregulated Exo-miRs in LS-*G6pc*^−/−^ mice with amyloidosis versus LS-*G6pc*^−/−^ mice without amyloidosis. Among these, some Exo-miRs were also found to be deregulated in the other groups analyzed (Table 1). In fact, miR-192-5p and miR-409-3p were also upregulated and miR-486a-5p was downregulated in LS-*G6pc*^−/−^ mice compared with WT (Table 1). Interestingly, miR-192-5p, miR-345-5p, miR-409-3p, miR-21a-5p and miR-150-5p have been associated with an inflammatory condition, and, among them, miR-345-5p, miR-21a-5p and miR-150-5p are unique to the amyloidosis status of the animals.

Therefore, the altered expression of several Exo-miRs is correlated with various pathologic liver conditions and might help to discriminate affected animals during the progression of the disease and development of late GSD1a-associated complications.

### Time-course analysis highlights the age-dependent modulation of Exo-miR representation in LS-*G6pc*^−/−^ mouse exosomes

**Fig. 1.**
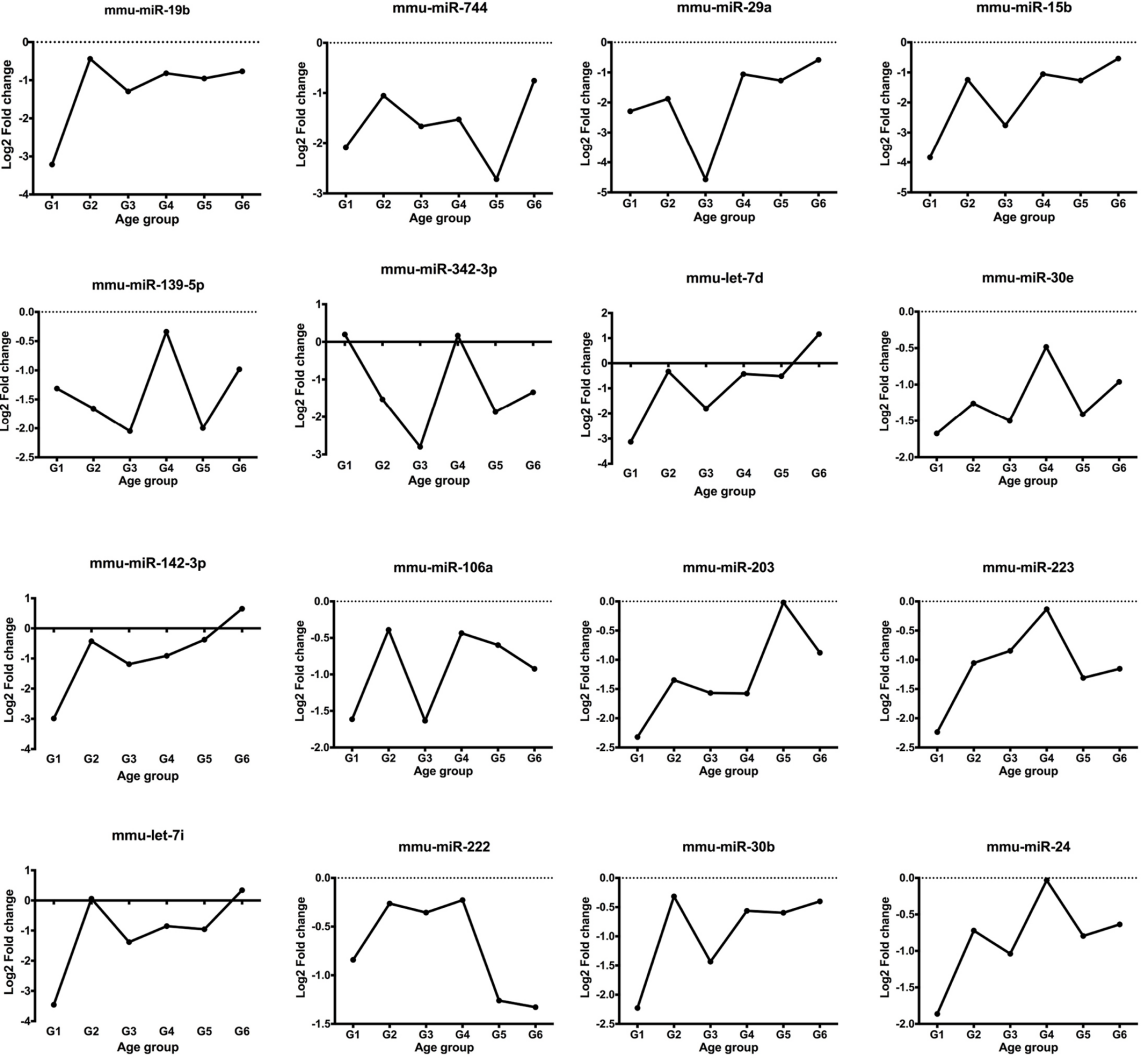
**Time course analysis reveals an age-dependent modulation of microRNA expression.** The plots show the median log2 fold change value for the 16 significant differentially represented Exo-miRs identified by the BETR method between LS-*G6pc*^−/−^ and WT mice grouped by age. The name of the microRNA is reported above each plot.

Understanding the trend of expression of candidate Exo-miRs could provide insight regarding the determination of biomarkers. Ideal biomarkers would not only be able to discriminate GSD1a patients from healthy subjects, but would also indicate the clinical stage. Therefore, the age-dependent modulation of Exo-miR representation might be instrumental to finding biomarkers of LS-*G6pc*^−/−^ mouse disease. The levels of the 61 Exo-miRs were examined in LS-*G6pc*^−/−^ mice at different time points during disease progression using the BETR method. The medians of their levels in both LS-*G6pc*^−/−^ and WT mouse exosomes were plotted at 1-3, 4-6, 7-9, 10-12, 12-15 and 16-18 months of age. The level of expression of 14 microRNAs was significantly reduced in LS-*G6pc*^−/−^ mice compared with WT mice in all age groups (Fig. 1). miR-744-5p, miR-29a-3p, miR-15b-5p, miR-342-3p and let-7i-5p were among the most significantly downregulated microRNAs when comparing LS-*G6pc*^−/−^ and WT mice. Furthermore, the levels of expression of let-7d and miR-142-3p in LS-*G6pc*^−/−^ mice increased over time, starting from a downregulation in younger LS-*G6pc*^−/−^ mice and becoming an upregulation in the older LS-*G6pc*^−/−^ mice. These findings indicate an age-dependent modulation of expression as the LS-*G6pc*^−/−^ mice became older.

### Validation of microRNAs by qPCR

To verify our findings, Exo-miRs extracted from plasma of LS-*G6pc*^−/−^ mice and WT mice were analyzed by individual quantitative PCRs (qPCRs) based on specific TaqMan microRNA assays. Ten different Exo-miRs, differentially represented in LS-*G6pc*^−/−^ mice versus WT mice or in LS-*G6pc*^−/−^ mice with HCA versus LS-*G6pc*^−/−^ mice without HCA, were validated. Correlation analysis was carried out by the Pearson correlation and linear regression analysis between RT-qPCR and array cards *Ct* values to assess the reproducibility of our experiments. The results confirmed a high positive correlation of microRNA representation between the two experiments (*r*>0.48 and *P*<0.05; Fig. S3). Furthermore, we performed a differential expression analysis between LS-*G6pc*^−/−^ mice and WT mice using Student's unpaired *t*-test on the *Ct* values generated with array cards. The *Ct* value was significantly higher for LS-*G6pc*^−/−^ mice than for WT mice, confirming the downregulation of these microRNAs (*P*<0.05; Fig. S4). These results validate the data of the microRNA analysis performed.

### Pathway analysis reveals the enrichment of insulin, chemokine, hypoxia and Wnt pathways in the profile of LS-*G6pc*^−/−^ mouse exosomes

We performed a pathway analysis based on microRNA target genes using gene ontology (GO) processes and Kyoto Encyclopedia of Genes and Genomes (KEGG) pathway anthologies. Pathway analysis was carried out for each significant Exo-miR (Table 1) using the MirWalk tool. Each significantly enriched pathway was associated with its regulating microRNA. For each set of Exo-miRs, the enriched pathways and processes were collected and organized into a functional enrichment results table. For the set of Exo-miRs significantly modulated in LS-*G6pc*^−/−^ versus WT mice, MirWalk identified 8384 targets. Pathway analysis showed significant enrichment of 32 GO biological processes and 62 KEGG pathways (adjusted *P*-value <0.05; Table S1). Among them, we observed an enrichment of target genes associated with AMPK and the insulin signaling pathway and thus with glucose and lipid metabolism for both miR744-5p and miR342-3p. Moreover, the enrichment of target genes involved in diabetes and its complications was associated with miR 744-5p. miR 342-3p and miR let-7i-5p modulate genes associated with cancer pathways and, among them, many genes linked to the Wnt/β-catenin pathway. In addition, two microRNAs targeting genes involved in chemokine signaling pathways (miR-744-5p), differentiation and activation of myeloid and lymphoid cells (let-7i-5p) were downregulated (Table 2).

**Table 2.**
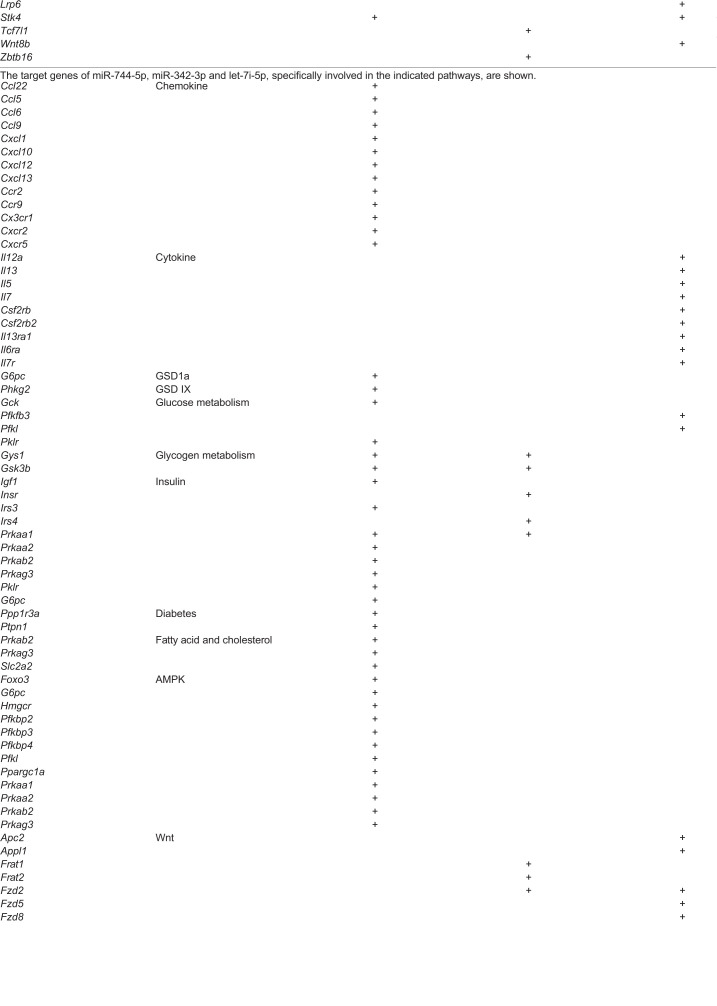
**Target **genes** of **miRs** significantly modulated in LS-*G6pc*^−/−^ mice**

For the set of Exo-miRs significantly modulated in LS-*G6pc*^−/−^ mice with HCA versus LS-*G6pc*^−/−^ mice without HCA, MirWalk identified 2605 targets. Pathway analysis showed significant enrichment of 34 GO biological processes and 16 KEGG pathways (adjusted *P*-value <0.05; Table S2). In particular, we observed an enrichment of miR-192-5p target genes involved in telomere maintenance and hepatocellular carcinoma and in the signaling pathway of FOXO1, a transcription factor that plays important roles in the regulation of gluconeogenesis and glycogenolysis by insulin signaling. Moreover, miR-192-5p targets genes involved in insulin resistance, apoptosis and immune regulation (Table 3).

**Table 3.**
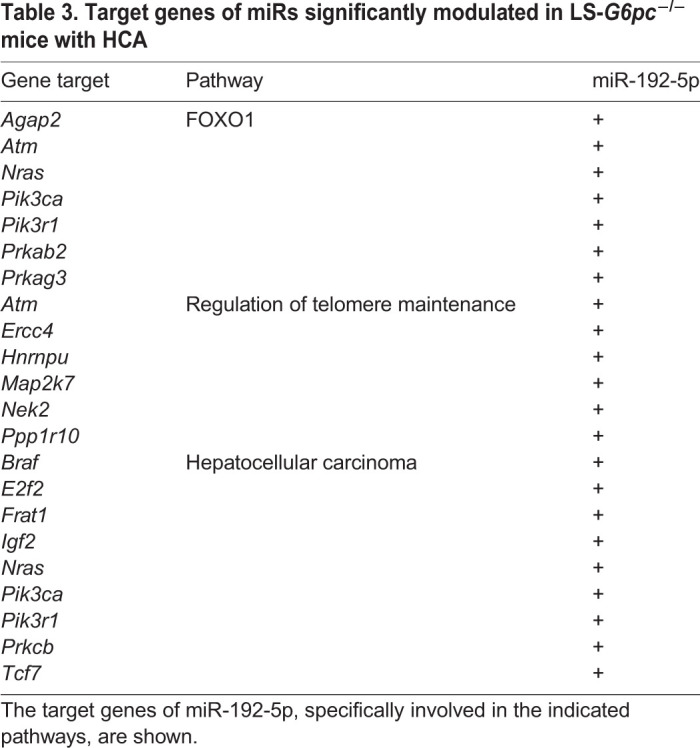
**Target genes of miRs significantly modulated in LS-*G6pc*^−/−^ mice with HCA**

For the set of Exo-miRs significantly modulated in LS-*G6pc*^−/−^ mice with amyloidosis versus LS-*G6pc*^−/−^ mice without amyloidosis, MirWalk identified 5995 targets. Pathway analysis showed significant enrichment of 51 GO biological processes and 64 KEGG pathways (adjusted *P*-value <0.05; Table S3). In particular, we observed an enrichment of miR-345-5p target genes involved in cancer pathways and hepatocellular carcinoma (Table 4). Of these, many genes belong to the Wnt/β-catenin signaling pathway, the processes of apoptosis or are associated with transcriptional regulation by *Hif1a*, such as *Arnt*, *Arnt2*, *Cul2* and *Egln1*. In addition, miR-192-5p, miR-345-5p and miR-21a-5p target genes involved in the regulation of both innate and adaptive immune responses.

**Table 4.**
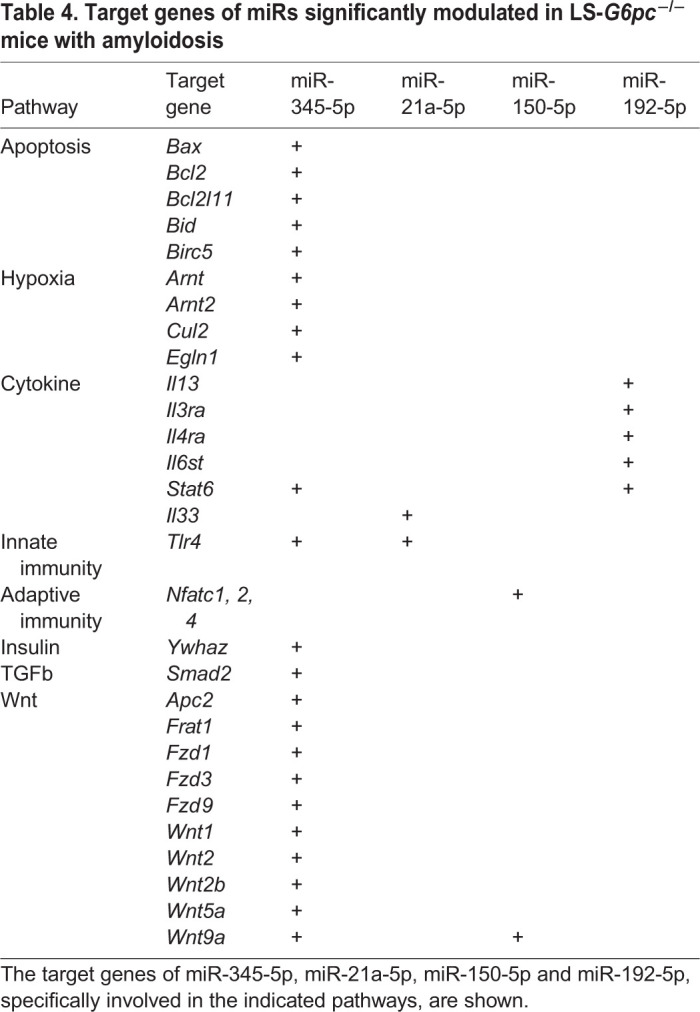
**Target genes of miRs significantly modulated in LS-*G6pc*^−/−^ mice with amyloidosis**

As reported above, 16 Exo-miRs showed modulation of expression over time. In particular, let-7d and miR-142-3p displayed a significant differential positive slope between LS-*G6pc*^−/−^ and WT mice. Pathway analysis carried out on let-7d and miR-142-3p showed significant enrichment of three GO biological processes and 38 KEGG pathways (adjusted *P*-value <0.05; Table S4). We again observed an enrichment of target genes involved in the Wnt/β-catenin and chemokine pathways (Table 5), suggesting that these pathways might be crucial in the progression of liver disease. let-7d also targets genes involved in inflammatory processes or chemokine signaling pathways.

**Table 5.**
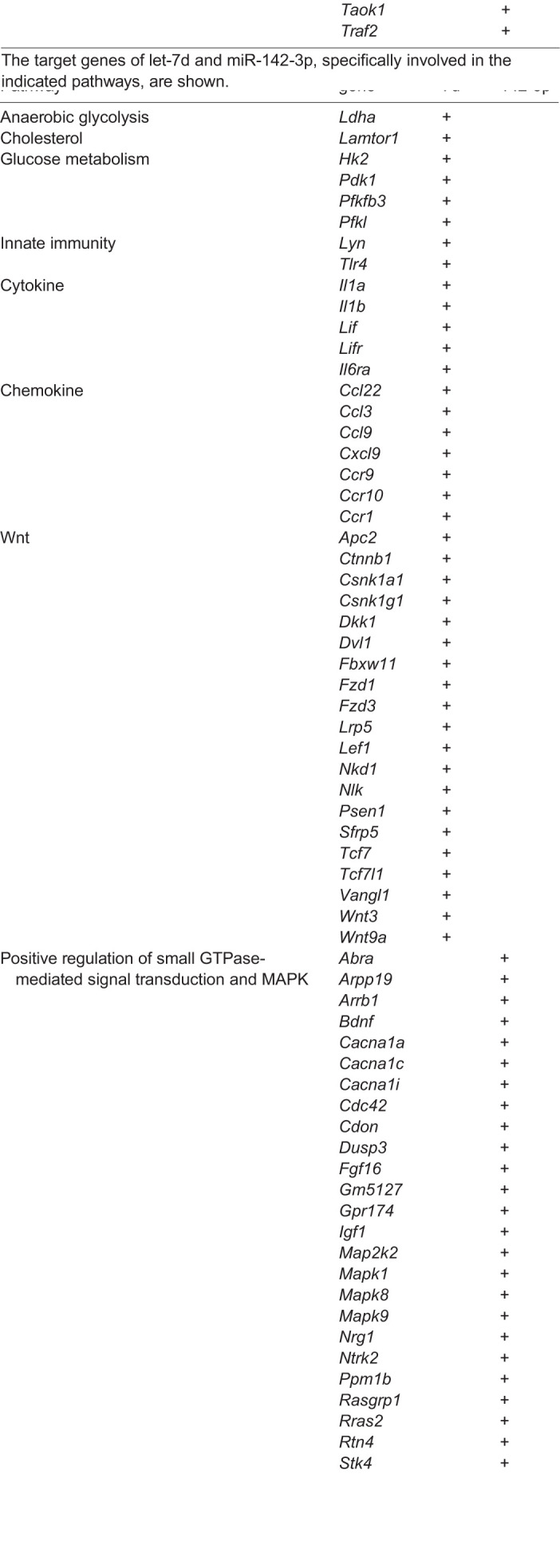
**Target genes of miRs significantly modulated in LS-*G6pc*^−/−^ mice over time**

### Analysis of overlapping between microRNA targets and Hallmark gene sets

**Fig. 2.**
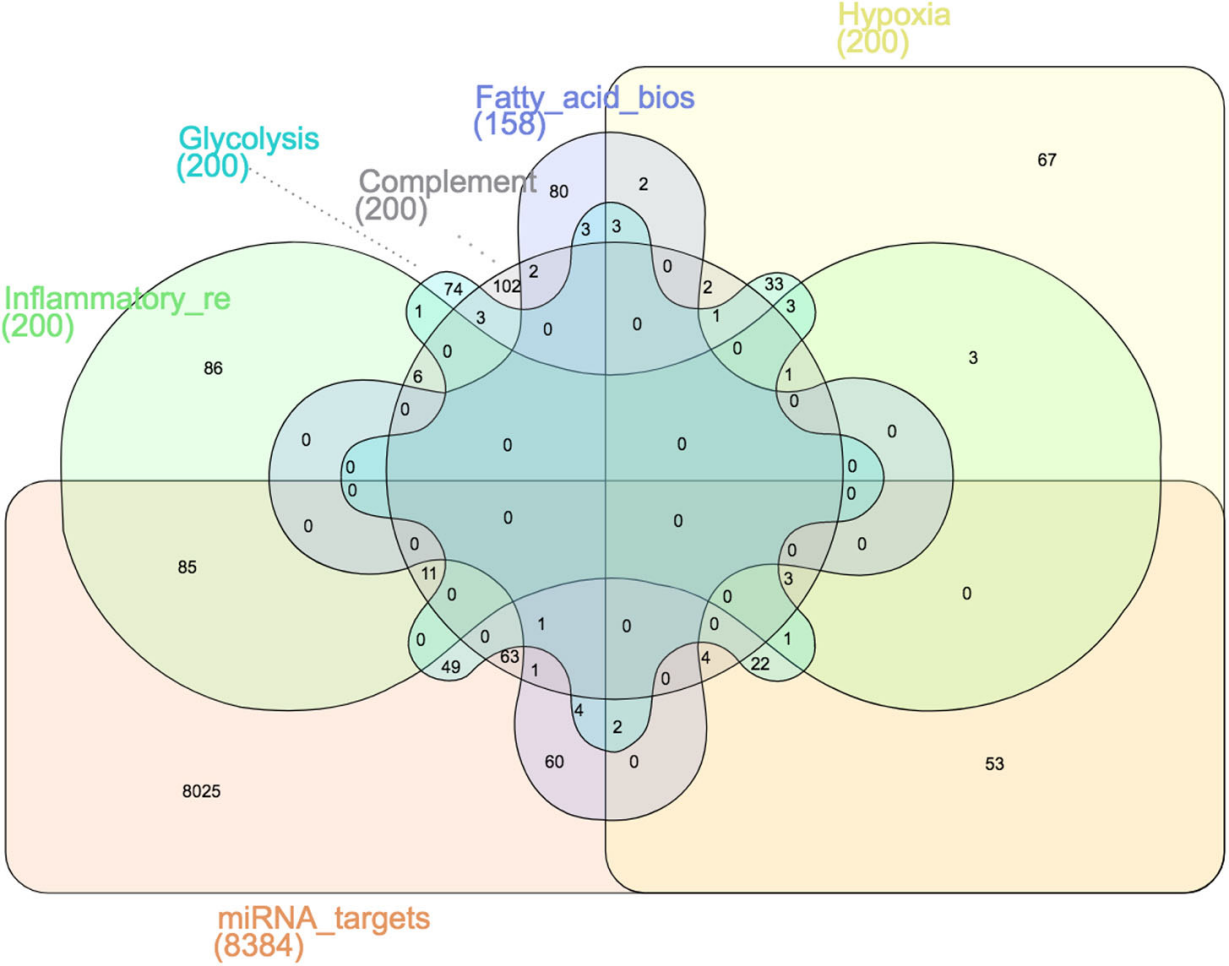
**Venn diagram among five selected hallmark gene sets.** Venn diagram showing exclusive and common genes among all possible combinations of five gene set enrichment analysis (GSEA) hallmark gene sets and Exo-miR target gene sets. Each combination is depicted by a distinct shape and color. Gray dotted lines link the gene set name and gene set shape when the gene set name could not be inserted above the shape. The GSEA gene sets used in the analysis belong to the hallmark collection of the Molecular Signature Database (MSigDB) v.6.2. A simplified colored name is reported for each MSigDB gene set for readability. The full MSigDB gene set names are as follows: HALLMARK_HYPOXIA, HALLMARK_GLYCOLYSIS, HALLMARK_FATTY_ACID_BIOSYNTHESIS, HALLMARK_INFLAMMATORY_RESPONSE and HALLMARK_COMPLEMENT. The number of genes of each MSigDB gene set is reported in parentheses below the gene set name. The targets of the Exo-miRs significantly modulated in LS-*G6pc*^−/−^ versus WT mice were organized into a gene set and referred to as microRNA targets. Exo-miR targets were identified using the MirWalk tool. The Venn diagram was drawn using the InteractiVenn tool.

Our recent study on the proteomic analysis of LS-*G6pc*^−/−^ and WT mouse livers (Cangelosi et al., 2019) reported evidence of metabolic reprogramming and a hypoxic environment within LS-*G6pc*^−/−^ mouse livers. On the basis of these findings, we hypothesized that exosomal microRNA might contribute to the regulation of these important biological conditions in LS-*G6pc*^−/−^ mice. We studied the overlapping between five known hallmark gene sets (glycolysis, hypoxia, fatty acid biosynthesis, inflammatory response and complement), retrieved from the MSigDB database version 6.2, and the targets of the microRNAs significantly modulated between LS-*G6pc*^−/−^ and WT mice. The five hallmark gene sets were chosen based on the results obtained by proteomic analysis. A Venn diagram between each Hallmark gene set and the 8384 microRNA targets is shown in Fig. 2. Hypergeometric tests performed on the overlapping genes reported significant overlap (*P*<0.05; Fig. 2). Furthermore, on analyzing the overlap between each couple of hallmark gene sets, we found that HALLMARK_HYPOXIA and HALLMARK_GLYCOLYSIS were the gene sets with the highest overlap (65 of 200 genes, 32.5%). This indicates that genes that contributed the most to enrichment were exclusive across gene sets, excluding the possibility that the overlap between hallmark gene sets and microRNA targets was constituted by the same set of genes.

This analysis reveals that Exo-miRs that are significantly modulated in LS-*G6pc*^−/−^ mice versus WT mice regulate genes connected with biological pathways previously identified by proteomic analysis as being associated with reprogramming of glucose-6-phosphate and with tumor development and progression.

## DISCUSSION

GSD1a animal models mirroring the human pathology are indispensable for basic and translational research aimed at discovering specific targets for the development of effective new therapies. The development of HCA in LS-*G6pc*^−/−^ mice is the consequence of a single gene mutation that inhibits the functioning of G6Pase-α and leads to liver degeneration and tumorigenesis. Thus, LS-*G6pc*^−/−^ mice are of major interest because they mimic spontaneous HCA formation with aging and its evolution into HCC, as in GSD1a patients. Thus, our animal model can provide an efficient means of discovering diagnostic markers of tumor formation in livers, and mouse and human cancer might have similar molecular signatures.

In this work, we evaluated whether Exo-miRs might represent potential biomarkers of GSD1a progressive pathologic manifestations, onset of liver tumors and development of HCC. An advantage of using the LS-*G6pc*^−/−^ mice was the possibility of performing the analysis in a homogeneous model, with a common genetic background and uniform disease progression. Moreover, the mouse model selected has allowed the clear definition of the hepatic contribution to the Exo-miR profile, because the liver is the only organ affected by the deletion of G6Pase-α in these mice. This initial approach allowed us to identify and select a restricted number of Exo-miRs whose expression is modulated in LS-*G6pc*^−/−^ mice.

Deregulation of distinct microRNAs in GSD1a patients with HCA was reported for the first time by Chiu et al. (2014). These authors proposed miR-130b as a new circulating biomarker for detection of GSD1a HCA. No overlap between the results obtained by Chiu et al. (2014) and our results was found except for miR-21, which we found to be upregulated in mice with amyloidosis and not in mice with HCA. The reason for the different results obtained in the two studies is probably attributable to the different experimental approaches. Chiu et al. (2014) conducted their study on liver or serum samples from GSD1a patients and healthy donors and compared the expression of selected microRNAs of GSD1a HCA samples with that of HCC cell lines, whereas we analyzed miR expression in circulating exosomes of mice and compared animals of similar age for each condition. In this respect, tissue might be more heterogeneous and possibly more prone to variations in expression of biological markers than exosomes, and mice might be more homogeneous than patients in the pathologic manifestations during the progression of the disease. Moreover, some studies have shown that there might be no correlation between serum microRNA and exosomal microRNA expression levels, probably because of RNase in serum, meaning that these two different assays cannot be substituted for each other (Zhang et al., 2019c), whereas in other cases differences between cancer tissues and exosomal microRNAs have been reported (Warnecke-Eberz et al., 2015).

The Exo-miRs we found to be deregulated in GSD1a mice with tumors are potentially relevant for the disease pathophysiology, according to the bioinformatic and literature analysis (Chen et al., 2018; Gao et al., 2017; Li et al., 2018; Zhang et al., 2019a). The signatures we have derived represent the liver-specific contribution to the Exo-miR profile of HCA development. We assume that the Exo-miRs differentially expressed between LS-*G6pc*^−/−^ and control mice that have been reported previously as biomarkers of HCC or have been involved in HCC development or progression can be regarded as prognostic markers of HCC development. Further analysis might allow a correlation between the Exo-miR profile and bHCA, of particular prognostic relevance for the development of HCC. Among the deregulated Exo-miRs, several are particularly attractive, because they have already been proposed as biomarkers for liver diseases and liver tumorigenesis (Liu et al., 2018; Tan et al., 2015; Wang et al., 2018a,b; Zhang et al., 2018) and might allow the identification of specific ‘signatures’ of HCA onset and disease progression, outcome or response to therapies. It was shown that miR-29a is downregulated in HCC and that this is correlated with overexpression of claudin, a protein involved in cell migration and metastasis (Mahati et al., 2017). miR-29a suppresses growth and migration of HCC by regulating CLDN1 (Mahati et al., 2017) and the oncogene IGF1R (Wang et al., 2017). miR-145-5p is involved in HCC-associated signaling pathways, such as Wnt, TGFβ and Ras, interacts with circular RNA in HCC (Qiu et al., 2019) and is one of the integrated signature of 13 microRNAs identified in HCC (Shi et al., 2015). miR-342-3p, like miR-29a, inhibits IGF1R (Liu et al., 2018) and might serve as a biomarker for poor prognosis in HCC (Gao et al., 2017). miR-744 was identified as an independent predictor of poor prognosis in HCC (Tan et al., 2015). miR-15b-5p inhibits OIP5, an oncogenic protein regulating cell cycle progression, which was found to be upregulated in HCC (Li et al., 2017) and is considered to be a biomarker for diagnosis of HCC (Chen et al., 2015). Finally, miR-142-3p is a tumor suppressor that inhibits HCC cell invasion and migration (Fu et al., 2018; Tsang et al., 2015) and might therefore represent another diagnostic biomarker for HCC patients.

**Fig. 3.**
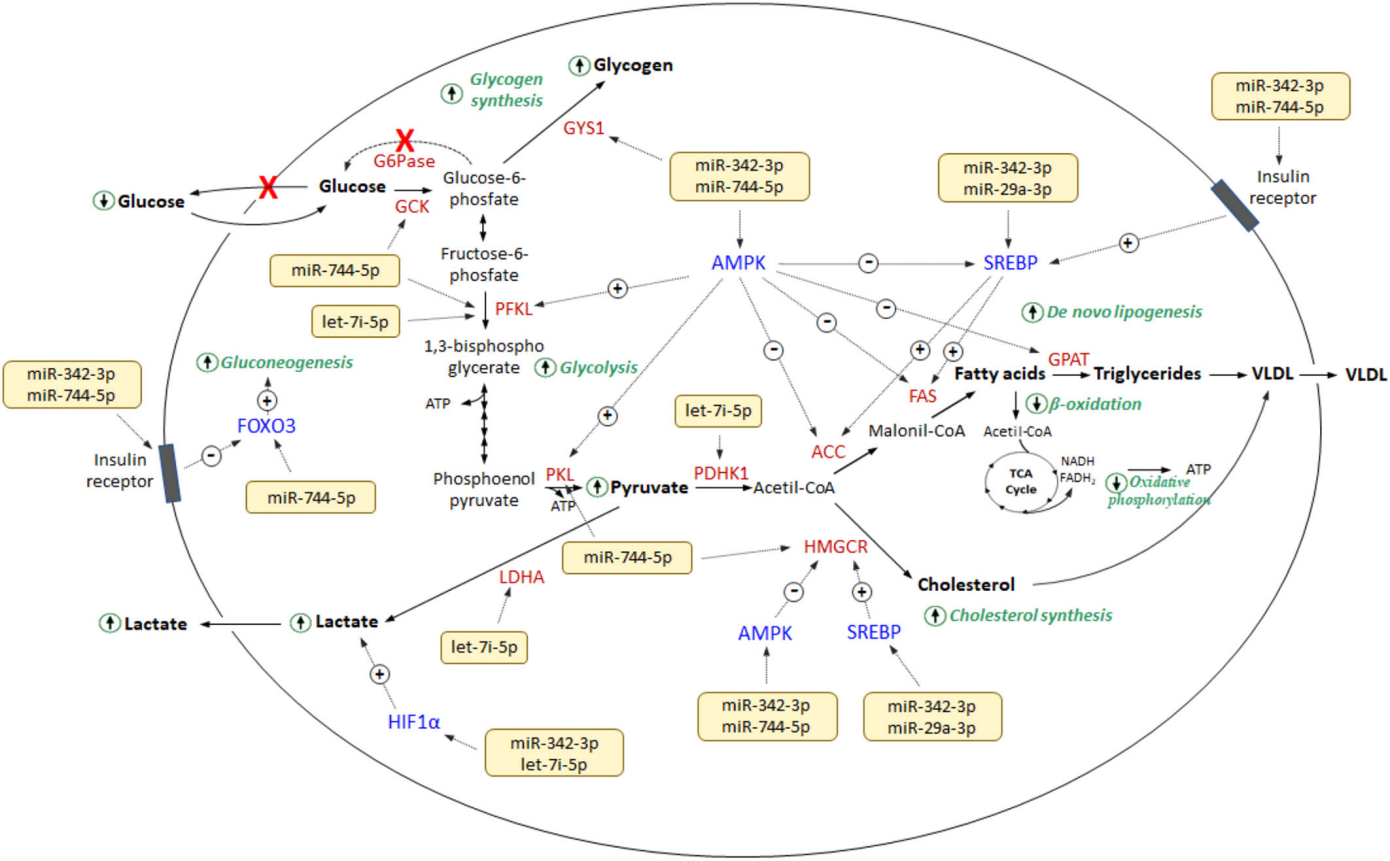
**Diagram showing the interaction among the deregulated miRs and targets relevant in glucose and lipid metabolism.**

Four of the seven downregulated Exo-miRs (i.e. let-7i-5p, miR-29-3p, miR-342-5p and miR-744-5p) are reported as direct or indirect modulators of the expression of multiple genes and/or signaling pathways (insulin signaling pathway and AMPK signaling pathway) relevant in glucose and lipid metabolism, suggesting their involvement in the regulation of energy metabolism reprogramming and the hypoxic conditions that characterize LS-*G6pc*^−/−^ liver versus WT (Fig. 3). In particular, miR-342-5p and miR-744-5p might upregulate hepatic glucose and glycogen levels by: (1) controlling, directly or via AMPK, glycolytic enzymes, including glucokinase (GCK), phosphofructokinase (PFK) and pyruvate kinase (PKL) (Liu et al., 2018; Zhang et al., 2019b); (2) regulating gluconeogenesis through the FOXO3 signaling pathway (Liang et al., 2013); and (3) controlling glycogen synthesis, owing to their action on glycogen synthase 1 (GSK1). miR-342-5p and miR-29a might be involved, via AMPK and/or SREBP, in the upregulation of cholesterol synthesis, through inactivation of HMG-CoA reductase (HMGCR) by phosphorylation or gene induction, respectively, and in *de novo* lipogenesis, via inactivation of acetyl-CoA carboxylase (ACC) and fatty acid synthase (FAS) by AMPK and overexpression by SREBP, resulting in increased amounts of fatty acids and triglycerides (Cheng et al., 2018; Li et al., 2013). In addition, the activity of ACC, FAS and HMGCR can be altered by miR-744-5p expression levels via the AMPK signaling pathway. Finally, let-7i-5p can also contribute to the reprogramming of metabolism by acting directly on lactate dehydrogenase A (*Ldha*) gene expression, resulting in the overproduction of lactate (Zhang et al., 2019b; Zhu et al., 2011). Lactate concentrations can be modified by miR-342-3p and let-7i-5p owing to their action on Hif1a expression (Zhang et al., 2017).

Chronic inflammatory conditions can be associated with systemic amyloidosis, a serious, rare pathologic complication. Amyloidosis is attributable to increased production of serum amyloid A (Saa) protein, a nonspecific acute phase protein synthesized in the liver under the control of pro-inflammatory cytokines. Increased Saa production can be induced by tissue damage, infection or abnormal pro-inflammatory cytokine activity. Saa is deposited on several organs as insoluble amyloid fibrils that can damage tissue functions, although it might take years of chronic inflammation before manifesting itself (Simons et al., 2013). We have observed marked amyloid deposition both in vessel walls and within sinusoids in the liver (Resaz et al., 2014) and in the kidney (R.R., L.M., F.G. and A.E., unpublished information) in 90% of LS-*G6pc*^−/−^ mice >10 months of age. These observations were confirmed by proteomic analysis revealing that serum amyloid P-component (Apcs), Saa1 and Saa2 were upregulated in the affected mouse livers (Cangelosi et al., 2019). The increased frequency of deposition of amyloid in LS-*G6pc*^−/−^ mice in comparison to humans affected by GSD1a might be attributable to the characteristics of these animals or, in general, to the difference in the lifespan of humans versus mice. In the present study, we found that several Exo-miRs were modulated in LS-*G6pc*^−/−^ mice affected by amyloidosis versus LS-*G6pc*^−/−^ mice without amyloidosis. Among them, miR-192-5p and miR-409-3p were also upregulated in LS-*G6pc*^−/−^ mice compared with WT, and miR-486a-5p was also downregulated in LS-*G6pc*^−/−^ mice versus WT (Table 1). Moreover, miR-192-5p, miR-345-5p, miR-409-3p, miR-21a-5p and miR-150-5p have been associated with inflammatory states, and, among them, miR-345-5p, miR-21a-5p and miR-150-5p are unique to the amyloidosis condition of the animals. Interestingly, miR-192-5p, miR-345-5p and miR-21a-5p can target genes involved in the regulation of both innate and adaptive immune responses. Another Exo-miR, let-7d, targets genes involved in inflammatory processes or chemokine signaling pathways. This microRNA is downregulated in LS-*G6pc*^−/−^ mice in comparison to WT mice.

The results indicate that LS-*G6pc*^−/−^ mice have an Exo-miR signature indicating both a reduction of the mechanisms participating in the resolution of inflammation (Il4, Il13 and Il33) and increased activation of the pathways promoting inflammation (Bottazzi et al., 2018). The latter include Il1a, Il1b, lif, the Il6 receptor (Il6ra) and different chemokines or chemokine receptors. Interestingly, Il6 (Bergmann et al., 2017) and Il1 (Sakurai et al., 2008) have been shown to contribute to the development of HCA and HCC. LS-*G6pc*^−/−^ mice also present microRNAs regulating genes involved in myeloid or lymphoid differentiation (Csf2rb, Il3 and Il7), the latter including T, B and innate lymphoid cells (ILCs) (Annunziato et al., 2015). These microRNAs also target genes coding for molecules involved in the recruitment of immune cells in peripheral tissues and for Il13 and Il5, cytokines known to promote type 2 immune responses and the development of chronic inflammation (Annunziato et al., 2015). In this context, a type 2-polarized microenvironment has been detected in most tumors, including HCC.

## MATERIALS AND METHODS

### Sample collection, histology and phenotype analyses

All animals were maintained in a conventional animal facility in 12 h-12 h light-dark cycles, fed *ad libitum* and monitored throughout their lifespan. All animal studies were approved by the Ethical Committee for Animal Experimentation (CSEA) as Animal Use project no. 291 communicated to the Italian Ministry of Health, with regard to the article of the D.lgs 116/92, and carried out at the animal facility of the National Institute for Cancer Research (Genova, Italy). Livers were fixed in 10% buffered formalin for 24 h. Formalin-fixed tissues were then processed, and 4-μm-thick sections were cut and stained with Hematoxylin and Eosin for histological analysis. All animals were evaluated phenotypically as described by Resaz et al. (2014). Sixteen LS-*G6pc*^−/−^ mice developed hepatic adenoma. Livers of WT mice were normal.

### Exosome isolation and microRNA purification from plasma

Blood was collected with a syringe from the left ventricle at the time of sacrifice, placed into EDTA tubes and centrifuged at 750 ***g*** for 10 min at room temperature (RT) to collect plasma. Plasma was stored at −80°C to be used for exosome isolation. Samples were collected at different time points (1-3, 4-6, 7-9, 10-12 and 13-15 months) that reflect different stages of disease progression. Exosome isolation and RNA extraction were performed with the exoRNeasy Serum/Plasma Midi kit (Qiagen Italia, Milano, Italy), as recommended by the manufacturer. Briefly, to isolate exosomes, 100 µl of plasma was centrifuged at 16,000 ***g*** at 4°C to eliminate cellular debris. Supernatants were then mixed with one volume of XBP binding buffer, loaded onto the exoEasy spin column, and spun at 500 ***g*** for 1 min at RT. Exosomes, bound to the filter of the column, were washed with 3.5 ml of XWP Washing Buffer by centrifuging at 5000 ***g*** for 5 min at RT. The spin column was transferred to a new collection tube and centrifuged at 5000 ***g*** for 5 min at RT after the addition of 700 µl of QIAzol to the membrane, to lyse exosomes and proceed with RNA purification. RNA extraction was performed according to the manufacturer's instructions. Quality control evaluation was performed with the Agilent 2100 Bioanalyzer, using the Small RNA Assay (Agilent Technologies Spa, Cernusco sul Naviglio, Milano, Italy).

### Quantitative real-time PCR (qRT-PCR)

Exo-miRs were analyzed with the TaqMan Array Card Technology. Exo-miRs were reverse transcribed with the TaqMan microRNA Reverse Transcription Kit, using the Megaplex RT primers Rodent Pool A (Thermo Fisher Scientific, Monza, Monza e Brianza, Italy). Pre-amplification of complementary DNA was performed with TaqMan PreAmp Master Mix and Megaplex Pre-Amp primers Rodent Pool A. The pre-amplification product was diluted according to the manufacturer's instructions and used to perform microRNA profiling on the ViiATM 7 Real-Time PCR System. Briefly, 9 µl of the diluted pre-amplified product was mixed with 450 µl TaqMan Universal Master Mix II, No UNG (Thermo Fisher Scientific) and 441 µl of nuclease-free water. One hundred microliters of the PCR reaction mix was dispensed into each well of the TaqMan Array Rodent microRNA A card (Thermo Fisher Scientific), enabling the quantification of 381 microRNAs. The modulation of Exo-miR expression in LS-*G6pc*^−/−^ mice was validated for five microRNAs by qRT-PCR on the ViiATM 7 Real-Time PCR System. Briefly, 0.3 µl of the diluted pre-amplified product was mixed with 7.5 µl TaqMan Universal Master Mix II, No UNG (Thermo Fisher Scientific), 1.75 µl of nuclease-free water and 0.75 µl of primers specific for each microRNA. The Δ*Ct* obtained with the array card was compared with that obtained by qRT-PCR to confirm differential expression if microRNA in every group analyzed.

### Bioinformatic procedures and statistical analysis

Data processing, categorization, normalization, filtering, imputation and differential expression were carried out using the PIPE-T galaxy tool (Zanardi et al., 2019). The *Ct* values falling within the range 14-32 were categorized as reliable values as recommended by the guidelines of the manufacturer. Global mean normalization was used to reduce any technical variability introduced in the data by the RT-qPCR experiments (Zanardi et al., 2019). Only Exo-miRs with ≤5% of missing values were retained for the analysis to reduce the bias introduced by imputation. The Mestdagh method (Zanardi et al., 2019) was used to assign a numeric expression value to missing values. The rank product method (Zanardi et al., 2019) implemented with the RankProd R package was used to identify significant differentially expressed microRNAs. Pathway analysis was performed for both predicted and validated targets of an Exo-miR using mirWalk version 3.0 (Sticht et al., 2018) and carried out using GO and KEGG gene set collections. An additional enrichment analysis was carried out using hallmark gene sets taken from MSigDB version 6.2 (Liberzon et al., 2015). The list of targets associated with an Exo-miR was identified by setting mouse as the species type. For time course analysis, mice were grouped according to their age into six groups, from 1 to 18 months of age, 3 months apart, and the analysis was carried out using the BETR R package (Aryee et al., 2009). To control the expected number of false-positive findings, we set up a maximum false discovery rate (FDR) of 5%. In order to focus on the most reliable age-dependent modulated Exo-miR, we considered an Exo-miR to be significant if the differential expression probability was >0.7. Overlapping among the lists of genes was carried out with Venn diagrams. The significance of the overlapping was estimated with hypergeometric statistics using the Stats R package (http://www.R-project.org/). The degree of correlation between the *Ct* values of RT-qPCR array cards and of validation experiments was assessed with the Pearson correlation. The significance of the difference of expression between LS-*G6pc***^−^**^/**−**^ and WT mice or between LS-*G6pc***^−^**^/**−**^ mice with HCA and those without HCA in the validation experiments was calculated using Student's unpaired *t*-test. Statistical analysis was carried out using GraphPad Prism version 6.0 for Mac (www.graphpad.com).

## Supplementary Material

Supplementary information
