# Supplementary material for: Circulating exosomal microRNAs as potential biomarkers of hepatic injury and inflammation in a murine model of glycogen storage disease type 1a
Source: Dis Model Mech. 2020 Sep 18;13(9):dmm043364. doi: 10.1242/dmm.043364 (PMC7520457; doi:10.1242/dmm.043364)
Supplement: Supplementary information [file dmm-13-043364-s1.pdf]

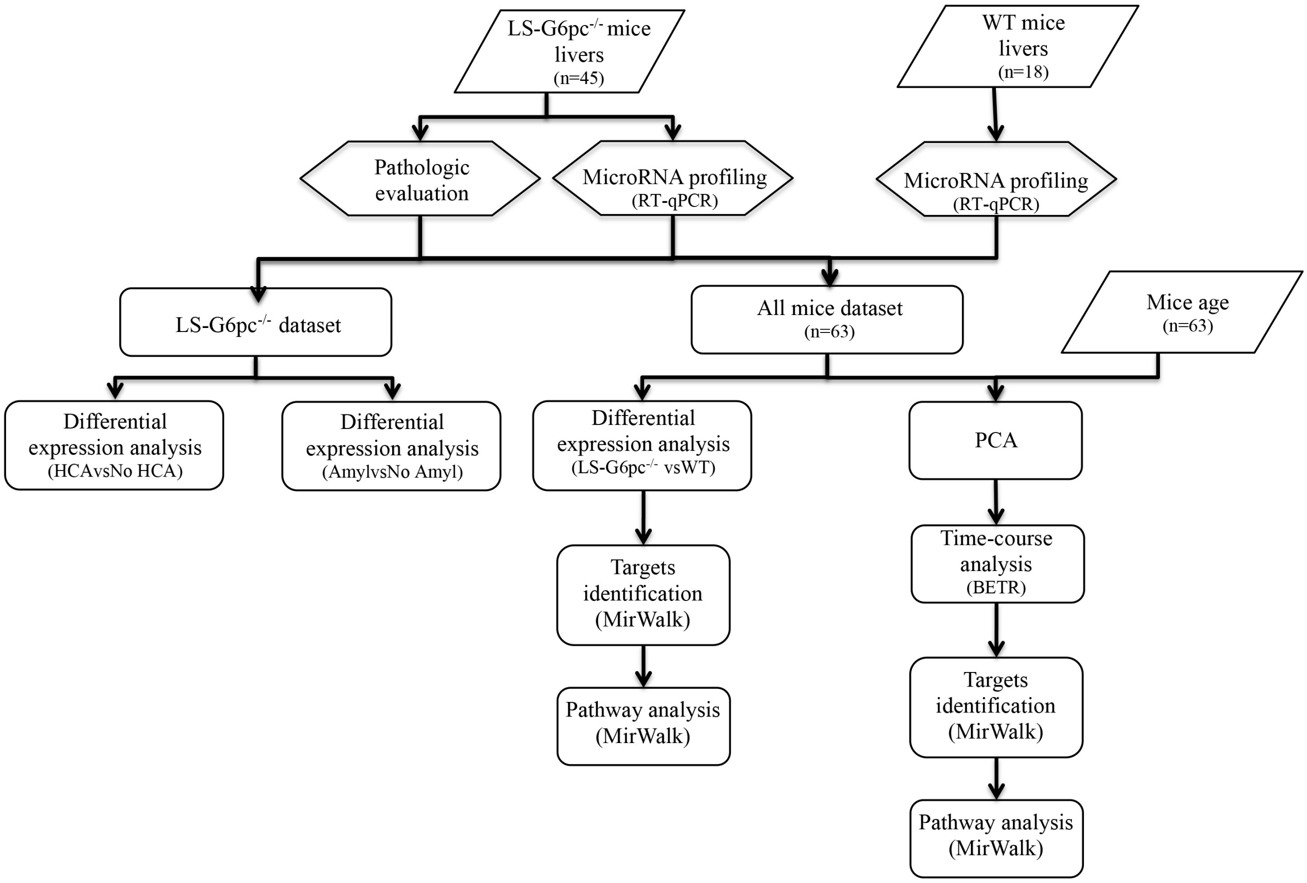

Fig. S1. Schematic representation of the whole bioinformatic strategy used in the study. The microRNA representation profile of 45 LS-G6pc$^{-/-}$ and 18 WT mouse plasma exosomes was measured by ViiA 7 RT-qPCR. Pathological evaluation of the livers assessed the presence of hepatic adenomas and/or amyloidosis within the entire set of mice samples. Differential expression analysis assessed any significant modulation of the Exo-miR between groups of LS-G6pc$^{-/-}$ and WT mice, LS-G6pc$^{-/-}$ mice characterized by the presence\absence of hepatic adenomas, or LS-G6pc$^{-/-}$ mice characterized by the presence/absence of amyloidosis. The representation profile of LS-G6pc$^{-/-}$ and WT mice over 6 distinct age groups was compared using BETR method. Analysis identified age-dependent modulated Exo-miR whose targets were identified using MirWalk tool. Pathway analysis on these targets identified the most significantly altered biological processes and pathways.

Disease Models & Mechanisms • Supplementary information

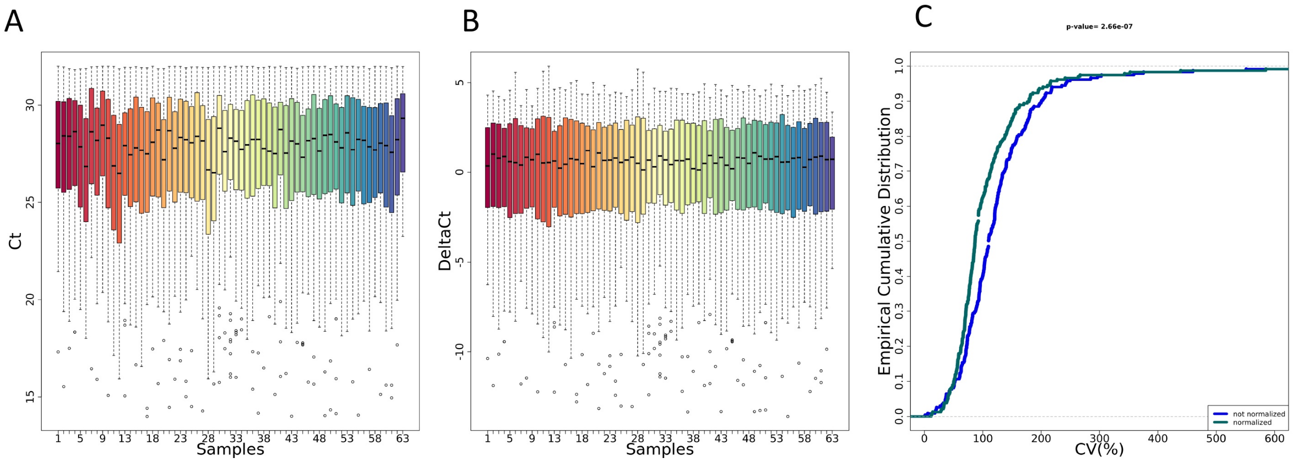

Fig. S2. Qualitative and quantitative assessment of the noise reduction for LS-G6pc$^{-/-}$ and WT mice data

The box plots reported in panels A and B show the distribution of Ct and delta Ct values for every samples before and after data normalization, respectively. The plot reported in panel C show the ECDFs (y axis) and the coefficient of variation (CV) for every samples before (blue line) and after (Green line) data normalization. Kolmogorov-Smirnov test assessed the significance of the separation between the curves and the p-value is reported on top of the plot. P-value lower than 0.05 is considered significant. Global mean was used to normalize the data.

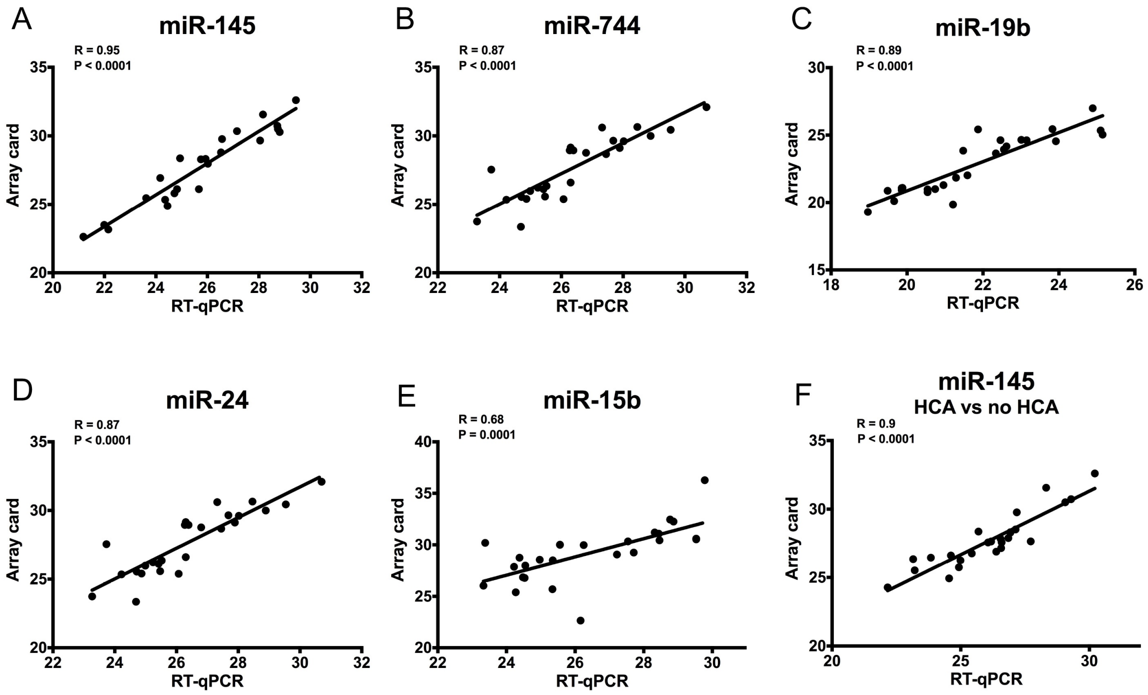

Fig. S3. Scatter plots of Ct values measured by RT-qPCR and Array card

A scatter plot showing correlation between the expression values measured by RT-qPCR and Array card on LS-G6pc$^{-/-}$ and WT mice samples is depicted for a selection of significant Exo-miR. Each point is the Ct value of a sample measured by RT-qPCR and Array card. Correlation is assessed by Pearson method. Linear regression line is superimposed to the points within each plot. Pearson correlation and p-value are reported in the top left side of the plots.

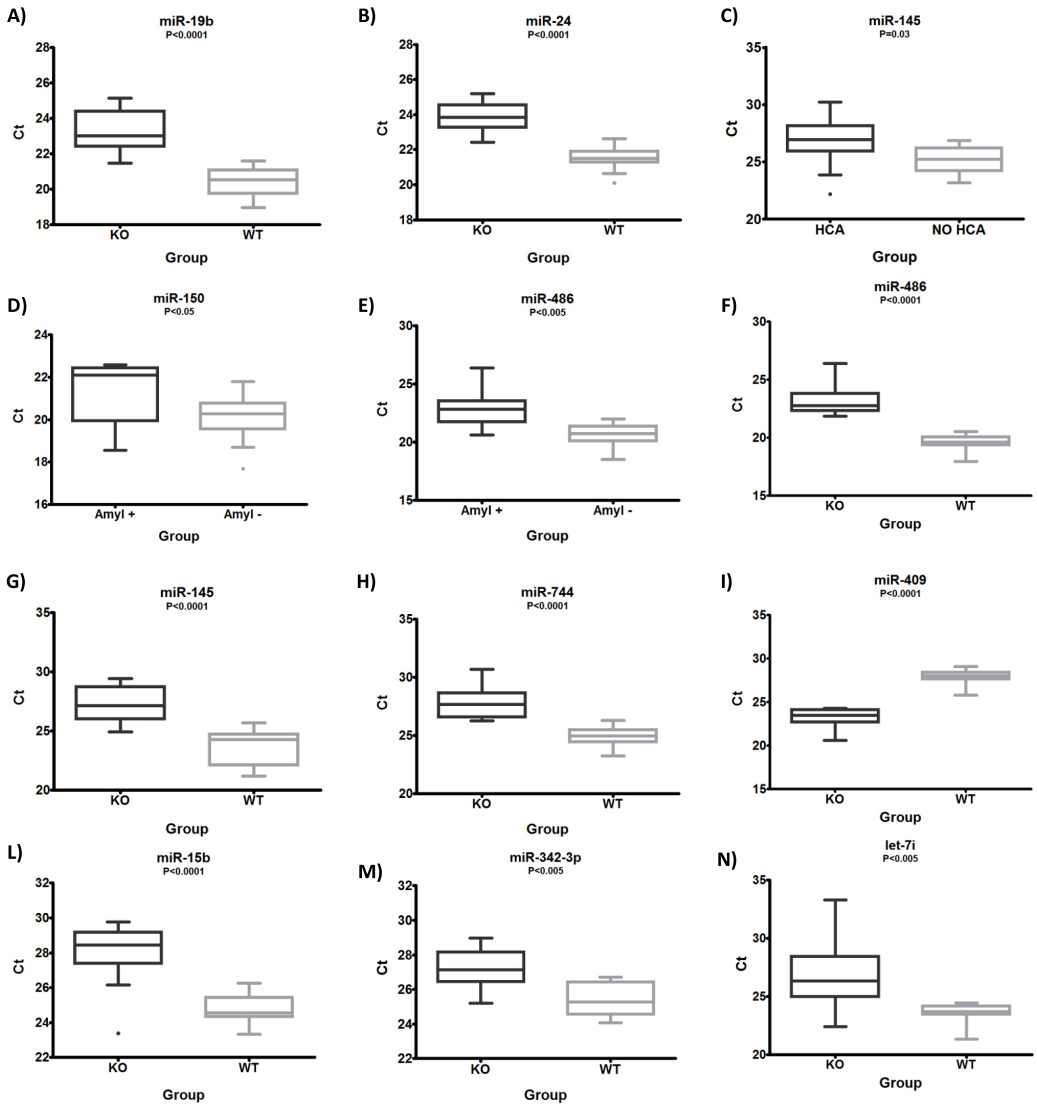

Fig. S4. Box plots of Ct values measured by RTqPCR between LS-G6pc$^{-/-}$ and WT mice; LS-G6pc$^{-/-}$ mice with and without amyloidosis; LS-G6pc$^{-/-}$ mice with and without adenoma.

Box-plots showing the differential expression between LS-G6pc$^{-/-}$ and WT mice, between LS-G6pc$^{-/-}$ mice with and without amyloidosis and between LS-G6pc$^{-/-}$ mice with and without adenoma are depicted for a selection of significant Exo-miR. Ct values were measured by RTqPCR. Statistical significance of the difference is assessed by unpaired t-test. P values and the name of the microRNA are reported on the top of each plot.

Disease Models & Mechanisms • Supplementary information

Table S1. Enrichment of GO biological processes and KEGG pathways in LS-G6pc$^{-/-}$ versus WT mice.

Click here to Download Table S1

Table S2. Enrichment of GO biological processes and KEGG pathways in LS-G6pc$^{-/-}$ mice with HCA versus LS-G6pc$^{-/-}$ mice without HCA.

Click here to Download Table S2

Table S3. Enrichment of GO biological processes and KEGG pathways LS-G6pc$^{-/-}$ mice with amyloidosis versus LS-G6pc$^{-/-}$ mice without amyloidosis.

Click here to Download Table S3

Table S4. Enrichment of GO biological processes and KEGG pathways in LS-G6pc$^{-/-}$ versus WT mice over time.

Click here to Download Table S4
